# Assessment of *Plasmodium falciparum* drug resistance molecular markers from the Blue Nile State, Southeast Sudan

**DOI:** 10.1186/s12936-020-03165-0

**Published:** 2020-02-18

**Authors:** Abdelrahim O. Mohamed, Maazza Hussien, Amal Mohamed, Abdelmaroof Suliman, Nuha S. Elkando, Hanadi Abdelbagi, Elfatih M. Malik, Mohammed H. Abdelraheem, Muzamil Mahdi Abdel Hamid

**Affiliations:** 1 Department of Biochemistry, Faculty of Medicine, University of Khartoum, Khartoum, Sudan; 2 Department of Medical Parasitology and Entomology, Faculty of Medical Laboratory Sciences, Al Neelain University, Khartoum, Sudan; 3 Institute of Endemic Diseases, Medical Campus, University of Khartoum, P. O. Box 102, Khartoum, Sudan; 4 Department of Accreditation, General Directorate of Quality, Development and Accreditation, Khartoum, Sudan; 5 State Ministry of Health, Blue Nile State, Damazin, Sudan; 6 Department of Community Medicine Faculty of Medicine, University of Khartoum, Khartoum, Sudan

**Keywords:** *P. falciparum*, Molecular marker, Sudan, *Pfcrt*, *Pfmdr1*, *Pfdhfr*, *Pfdhps*, *Pfk13*, *Exonuclease*

## Abstract

**Background:**

*Plasmodium falciparum* malaria is a public health problem worldwide. Malaria treatment policy has faced periodic changes due to emergence of drug resistant parasites. In Sudan chloroquine has been replaced by artesunate and sulfadoxine/pyrimethamine (AS/SP) in 2005 and to artemether–lumefantrine (AL) in 2017, due to the development of drug resistance. Different molecular markers have been used to monitor the status of drug resistant *P. falciparum*. This study aimed to determine the frequency of malaria drug resistance molecular markers in Southeast Sudan.

**Methods:**

The samples of this study were day zero dried blood spot samples collected from efficacy studies in the Blue Nile State from November 2015 to January 2016. A total of 130 samples were amplified and sequenced using illumina Miseq platform. The molecular markers included were *Pfcrt*, *Pfmdr1*, *Pfdhfr*, *Pfdhps*, *Pfk13*, *exonuclease* and artemisinin resistant (ART‐R) genetic background (*Pfmdr2*, *ferroredoxine*, *Pfcrt* and *Pfarps*10).

**Results:**

Resistance markers for chloroquine were detected in 25.8% of the samples as mutant haplotype *Pfcrt* 72-76 CVIET and 21.7% *Pfmdr1* 86Y. *Pfdhfr* mutations were detected in codons 51, 59 and 108. The **I**C**N**I double-mutant haplotype was the most prevalent (69%). *Pfdhps* mutations were detected in codons 436, 437, 540, 581 and 613. The S**GEG**A triple-mutant haplotype was the most prevalent (43%). In *Pfdhfr*/*Pfdhps* combined mutation, quintuple mutation **I**C**N**I/S**GEG**A is the most frequent one (29%). Six of the seven treatment failure samples had quintuple mutation and the seventh was quadruple. This was significantly higher from the adequately responsive group (P < 0.01). *Pfk13* novel mutations were found in 7 (8.8%) samples, which were not linked to artemisinin resistance. Mutations in ART‐R genetic background genes ranged from zero to 7%. Exonuclease mutation was not detected.

**Conclusion:**

In this study, moderate resistance to chloroquine and high resistance to SP was observed. Novel mutations of *Pfk13* gene not linked to treatment failure were described. There was no resistance to piperaquine the partner drug of dihydroartemisinin/piperaquine (DHA-PPQ).

## Background

Malaria is a major public health problem in the World. In 2017, the World Health Organization (WHO) estimated that 219 million people got malaria with death amounting to 435,000 mainly in Africa [1]. In the Sudan, 720,879 cases were reported as confirmed malaria and 1446 were reported as deaths in 2017. *Plasmodium falciparum* is the main malaria parasite species responsible for 92% of all malaria cases while, *Plasmodium vivax* represents approximately 8% [1].

Malaria treatment policy in Sudan has been changed through decades from chloroquine for the uncomplicated cases and quinine for complicated cases to sulfadoxine/pyrimethamine (SP) antifolate drugs to other monotherapies which all failed through time until the introduction of artemisinin-based combination therapy (ACT) in 2005 [2–4].

Chloroquine resistance emerged worldwide as early as late fifties in Southeast Asia and South America [5]. In Sudan, chloroquine resistance started in late 1970s, and treatment failure became alarmingly high until the introduction of ACT in 2005 [4, 6, 7].

Single nucleotide polymorphisms (SNPs) in *Pfcrt* and *Pfmdr1* genes have been correlated with chloroquine resistance for *P. falciparum* around the world [8, 9]. Mutations in these genes have been identified in several studies from different parts of the Sudan [7, 10, 11]. The resistant haplotypes of *Pfcrt* have been identified as 72–76 CVIET and *Pfmdr1* responsible for chloroquine resistance as 86Y [8, 9, 12]. SP was introduced in Sudan for the treatment of malaria in early 1970s as a second-line treatment with chloroquine as the first-line of treatment [6]. SP has gained excellent reputation as a combination therapy that targets two different sites of the folate metabolism pathway [13] and, therefore, was the first combination therapy used for malaria treatment in Sudan. It continued to be used in pregnancy and when chloroquine resistance became a problem. However, malaria parasites developed resistance to this combination as well [14]. Previous researches which were done in Sudan identified the responsible resistance genes for SP. The genes of resistance were mutants of dihydrofolate reductase (*dhfr* 51I, 59R and 108N) and mutants of dihydropteroate synthase (*dhps*436A, 437G, 540E and 581G) of the pyrimethamine and sulfadoxine, respectively [14–16].

Artemisinin-based combination therapy became the rescue rope for malaria treatment in Sudan where it has been introduced in 2005 by using AS/SP [4]. This combination together with other control measures remarkably reduced the cases of malaria in Sudan during 10 years period [1]. Again treatment failure to this combination became a public health problem [15, 17] and the combination of artemether/lumefantrine as first-line of treatment of uncomplicated malaria and dihydroartemisinin/piperaquine (DHA-PPQ) as a second-line were introduced in March 2017 [17]. *Exonuclease* gene mutation (415G) has been noticed to be associated with increased tolerance of piperaquine [18].

Quinine has remained to be the first choice for severe malaria and malaria in pregnancy in Sudan. However, there are some concerns about decreased efficacy of quinine [19]. Mutations in the kelch propeller protein gene (*k13*) have been used as markers for delayed clearance of *P. falciparum* by artemisinin derivatives. Several non-synonymous mutations in the propeller domain of the gene have been reported from different parts of the world. The identified alleles so far linked to reduced clearance of the parasite have been reported earlier [20]. Parasite genetic background mutations that allow for emergence of *Pfk13* mutations have been studied earlier [21], including *Pfmdr2*, *ferroredoxine*, *Pfcrt* and *Pfarps10*.

This research examined the different resistance markers for *P. falciparum* malaria from an area of unstable malaria transmission in southeastern Sudan. The main objective was to examine the resistance situation of all previously used drugs like chloroquine and the concurrent anti-malarial artemisinin-based combination therapy using molecular drug resistance markers.

## Methods

### Study area

The study was conducted in two health centres in Damazin, the Capital of the Blue Nile State, Southeastern Sudan (location 11.7855° N, 34.3421° E). Malaria transmission is seasonal unstable transmission following the rainy season which is July to October [17]. There is another minor peak during December to February winter months [22].

### Study samples and DNA extraction

The samples of this study were dried blood spots (DBS) from uncomplicated *P. falciparum* malaria patients taken at day zero. Samples were part of efficacy studies for AS/SP (n = 63) and DHA-PPQ (n = 67) performed in the Blue Nile state between November 2015 and January 2016, Sudan [17]. The total number of samples was 130 (123 were from the adequately responsive patients and 7 with late parasitological drug failure (LPF) belonging to AS/SP group). Among the group of samples for *Pfk13* gene sequencing, 28 were excluded because they were reported earlier [23]. The samples were collected after obtaining informed written consent from patients or the guardians of minor patients. The study received ethical clearance from the Federal Ministry of Health, Sudan.

DNA was extracted from 130 samples using QIAmp DNA Mini Kit (QIAGEN Inc., Germany) following the manufacturer’s instructions. DNA was eluted in 50 µl and stored at − 20 °C for use in the PCR assays.

### Plasmodium species confirmation

*Plasmodium falciparum* was identified by microscopic examination of Giemsa-stained slides. Furthermore, the presence of other *Plasmodium* species were checked by sequencing of two conserved regions of the *Plasmodium* parasite mitochondrial genome [24]. This procedure was done to confirm that the samples were only *P. falciparum*.

### Complexity of infection (COI)

Estimation of COI from SNP genotyping data was performed using the programs COIL [25] and Real MCOIL [26]. Both programs used the SNP barcode of 101 bi-allelic unlinked SNPs genotyped by amplicon sequencing (below). The COI is expressed as an integer, which is the estimated number of individual parasites within the sample.

### Amplicon sequencing

Amplicon sequencing of parasite DNA samples was performed at the Wellcome Sanger Institute, UK for genotyping of drug resistance markers (*Pfcrt, Pfmdr1*, *Pfdhfr*, *PfdhPfs*, *exonuclease*, *Pfk13* and artemisinin resistance (ART-R) genetic background, *Pfarps10*, *ferredoxin*, *Pfcrt*, *Pfmdr2*). Parasite genetic barcodes, and specification of the markers will appear in a manuscript in preparation by Wellcome Sanger Institute. In brief, targets for genotyping were identified and multiplex PCR primers were designed using a modified version of the mPrimer software. Primers were designed to amplify products between 190 and 250 bp and were combined into 3 pools. Before targeted amplification by PCR, a selective whole-genome amplification (sWGA) was done on extracted genomic DNA to increase the concentration of parasite DNA [27]. A two-step protocol was used to first amplify the target regions of the parasite genome, followed by a second PCR to incorporate sequencing and multiplexing adapters. PCR products were size selected and pooled into a single volume, and batched samples were sequenced in a single Illumina MiSeq lane. Samples reads were de-plexed using the multiplexing adapters and individual CRAM files were aligned to a modified amplicon Pf3D7 reference genome. Genotyping was done using bcf tools as well as custom scripts to filter and translate genotypes into drug resistance haplotypes [28]. Sequences were deposited in the public repository European Nucleotide Archive (ENA) with accession numbers provided as Additional file 1.

### Data analysis

Allele and genotype data were entered into SPSS software v. 20 and frequencies were calculated. A correlations between treatment response and SP genotypes was calculated. Chi^2^ test was used to calculate the significance.

## Results

Out of the 130 samples different numbers were successfully sequenced for the different genes.

### Complexity of infection (COI)

All samples were confirmed to be only *P. falciparum* parasites. Complexity of infection (COI) was detected in 39 (32%) isolates, where 34 (28%) contained 2 parasite clones per sample and 5 (4%) contained 3 clones per sample.

### Molecular markers for drug resistance

Genotyping of chloroquine resistance gene (*Pfcrt*) showed 31 (25.8%) mutant haplotypes at positions 72–76 (CVIET) (Table 1). Other mutations of *Pfcrt* are also shown (Table 1).

**Table 1:** Prevalence of *Pfcr*t haplotypes and alleles in *P. falciparum* isolates from Southeastern Sudan

*Pfcr*t haplotypes/alleles	Frequency n = 120
Wild type CVMNK	89 (74.2%)
Mutant type CVIET	18 (15%)
Multiple clones CV[M/I][N/E][K/T]	13 (10.8%)
M74I	31 (25.8%)
N75E	31 (25.8%)
K76T	31 (25.8%)
A220S	35 (29.1%)
Q271E	37 (30.8%)
R371I	35 (29.1%)

N86Y mutation of *Pfmdr1* was detected in 25 (21.7%) samples, while *Pfmdr1* Y184F was seen in 89.5% of the samples (Table 2).

**Table 2:** Prevalence of *Pfmdr*1, *Pfdhfr*, *Pfdhps*, *Pfk13*, ART‐R genetic background alleles in *P. falciparum* isolates from Southeastern Sudan

Drug resistance marker	Mutant alleles	Frequency
*Pfmdr*1 n = 115	N86Y	25 (21.7%)
	Y184F	103 (89.5%)
	D1246Y	2 (1.7%)
*Pfdhfr* n = 119	N51I	107 (89.9%)
	C59R	35 (30.4%)
	S108N	116 (97.4%)
*Pfdhps* n = 104	S436A	7 (6.7%)
	A437G	86 (83%)
	K540E	79 (76%)
	A581G	45 (43%)
	A613T/S	2 (1.9%)
*Pfk13* n = 79	F375S	1 (1.3%)
	K378R	1 (1.3%)
	D389N	1 (1.3%)
	K430K	1 (1.3%)
	E433D	1 (1.3%)
	P443P	1 (1.3%)
	N594K	1 (1.3%)
(ART‐R genetic background) n = 114	*Ferredoxine* D193Y	1 (0.87%)
	*Pfcrt* N326S	8 (7%)
	*Pfcrt* I356T	4 (3.5%)

Table 2 also shows mutations of *Pfdhfr* N51I which was detected in 107 (89.9%) samples, *Pfdhfr* S108N was seen in 116 (97.4%) samples. Alleles of *Pfdhps* are shown in Table 2 as well, where A437G was detected in 86 (83%) samples and 79 (76%) samples were K540E. There were no mutations detected in *exonuclease* E415G allele. *Pfdhfr* genotypes are shown in Table 3 where double mutations (**I**C**N**I) were seen in 69% of the samples, while triple mutations were seen in 21% (**IRN**I).

**Table 3:** Frequency of genotypes of Pfdhfr and *Pfdhps* among the AS/SP and DAPPQ groups *P. falciparum* isolates from Southeastern Sudan

Drug resistance marker	Number of mutations	Mutation haplotype	DAPPQ n = 55	AS/SP n = 49	Total (n 104) n (%)
*dhfr*	None	Wild type NCSI	3 (5.45%)	0 (0%)	3 (2.9%)
	Double	**I**C**N**I	40 (72.7%)	32 (65.3%)	72 (69.2%)
		N**RN**I	1 (1.8%)	6 (12.2%)	7 (6.7%)
	Triple	**IRN**I	11 (20%)	11 (22.4%)	22 (21.1%)
*dhps*	None	Wild type SAKAA	11(20%)	3 (6.1)	14 (13.5%)
	Single	**A**AKAA	4 (7.3%)	0 (0%)	4 (3.8%)
		S**G**KAA	4 (7.3%)	3 (6.1)	7 (6.7%)
	Double	S**GE**AA	15 (27.3)	17 (34.7%)	32 (30.7%)
	Triple	S**GEG**A	21 (38.1)	24 (48.9%)	45 (43.2%)
		**AGE**AA	0 (0%)	1 (2%)	1 (0.96%)
		S**GE**A**T**	0 (0%)	1 (2%)	1 (0.96%)
*dhfr *+ *dhps*	None	Wild type NCSI + SAKAA	1 (1.8%)	0 (0%)	1 (0.96%)
	Single	NCSI + **A**AKAA	2 (3.6%)	0 (0%)	2 (1.9%)
	Double	**I**C**N**I +SAKAA	8 (14.5%)	3 (6.1%)	11 (10.6%)
	Triple	**IRN**I +SAKAA	2 (3.6%)	0 (0%)	2 (1.9%)
		**I**C**N**I + **A**AKAA	3 (5.45%)	0 (0%)	3 (2.9%)
		**I**C**N**I + S**G**KAA	0 (0%)	2 (4%)	2 (1.9%)
		N**RN**I + S**G**KAA	1 (1.8%)	1 (2%)	2 (1.9%)
	Quadruple	**IRN**I + S**G**KAA	2 (3.6%)	0 (0%)	2 (1.9%)
		**I**C**N**I + S**GE**AA	14 (25.5%)	9 (18.4%)	23 (22.1%)
		N**RN**I + S**GE**AA	0 (0%)	1 (2%)	1 (0.96%)
	Quintuple	**IRN**I + S**GE**AA	1 (1.8%)	7 (14.3%)	8 (7.7%)
		**I**C**N**I + **AGE**AA	0 (0%)	1 (2%)	1 (0.96%)
		**I**C**N**I + S**GEG**A	15 (27.3)	16 (32.6%)	31 (29.8%)
		**I**C**N**I + S**GE**A**T**	0 (0%)	1 (2%)	1 (0.96%)
		N**RN**I I + S**GEG**A	0 (0%)	4 (8.1%)	4 (3.8%)
	Hextuple	**IRN**I + S**GEG**A	6 (10.9%)	4 (8.1%)	10 (9.6%)

Concerning *Pfdhps* (Table 3), triple mutations (S**GEG**A) showed the highest frequency (43.2%). All samples that showed mutation in codon A581 were mutant in codon K540E and A437G and samples that were mutated in codon K540E were also mutant in A437G.

Combination of *Pfdhfr* and *Pfdhps* mutations as seen in AS/SP and DHA-PPQ groups are shown in Table 3. The highest combined mutation was the quintuple mutation (**I**C**N**I + S**GEG**A) was 29.8% followed by the quadruple mutation (**I**C**N**I + S**GE**AA) (22.1%). Quintuple mutation was detected in 6 of the seven AS/SP drug failure isolates while the last one harboured quadruple mutation. A correlation between the adequately responsive group and the late parasitological failure group has shown that there is a significant association of the quintuple mutation with the failure group (P < 0.01).

*Pfk13* showed novel non‐synonymous mutations in 5 samples F375S, K378R, D389N, E433D, N594K and 2 samples showed synonymous mutations K430K, P443P (Table 2).

No mutations were detected in *Pfcrt* (C72S, H97Q), *Pfmdr1* (S1034C, N1042D, F1226), *Pfdhfr* (A16V, I164L), (ART‐R genetic background) *Pfarps10* (V127M, D128Y\H), *Pfmdr2* T484I.

## Discussion

This is a study of molecular markers for drug resistance genes of *P. falciparum* malaria from the Blue Nile State in Sudan. In this study, complexity of infection was observed in 32% of the samples indicating high transmission [29]. Different mutations of *Pfcrt* indicating resistance have been reported in this study with total of 25.8%. However, this percentage is low compared to other areas in the country where *Pfcrt* mutations ranging from 63 to 100% were reported [7, 11, 16, 30, 31]. Chloroquine resistance mutations is not reversible. However, when chloroquine pressure is removed sensitive strains dominate [32, 33] so the low percentage may indicate this phenomenon. This percentage of resistance is still high for reinstitution of chloroquine which requires resistance level not exceeding 10% [34].

Mutation of *Pfmdr1* N86Y is also a marker for chloroquine resistance (21.7%) is consistent with that of *Pfcrt*. Other reports in Sudan have shown higher levels of this mutation [7, 30, 31]. The allele Y184F showed high percentage of 89.5%, which favours the use of the first-line treatment AL as this mutation increases susceptibility of the parasite to lumefantrine [35, 36].

Reported mutations in *Pfmdr1* at positions 1034, 1042 and 1226 affect several anti-malarial drugs such as mefloquine, chloroquine, quinine, and halofantrine [36, 37]. There are no mutations in these alleles associated with resistance to these drugs in this study.

Resistance to amodiaquine is linked with the same mutation linked to chloroquine resistance *Pfmdr1* at positions 86 and 1246 [38, 39]. This needs more verification with in vivo studies for use of amodiaquine as a prophylactic drug.

DHFR and DHPS are the enzymes that metabolize antifolate drugs and mutations in their genes have been reported to cause treatment failure with antifolates pyrimethamine and sulfadoxine, respectively [40]. In this study, multiple mutations of *Pfdhfr* and *Pfdhps* are reported (Table 3). Mutant genotype combinations are mostly linked to increasing resistance from double to quintuple mutations [41]. In this study, quadruple and quintuple mutations represent 68%, hextuple mutations are also described in this study forming nearly 10%. The quintuple mutation (**I**C**N**I/S**GEG**A) is strongly linked with the treatment failure group. This finding indicates high level of resistance of SP. Earlier reports in Sudan have shown that multiple mutations were highly prevalent in eastern Sudan [7, 14]. Failure of combination therapy AS/SP in Sudan was ascribed to failure of the partner drug SP [23] and that combination was replaced by AL as the first-line of treatment [17]. This high resistance of SP also means that it cannot be used intermittently for the protection of pregnant women in highly endemic areas in Sudan.

*Exonuclease* gene mutation is one of the markers for resistance to piperaquine [18] and is reported to be zero in this study. The other markers were not studied. The choice of DHA–PPQ as second line of treatment [17] is well supported by this evidence. PPQ enjoys the fact that it has never been used as a mono-therapy for malaria treatment [42, 43].

Artemisinin-based combination therapy has revolutionized malaria treatment. Mutations associated with delayed clearance of parasites have been reported from Southeast Asia posing a challenge to the rest of the world. Close monitoring of mutations in the propeller protein *k13* gene of *P. falciparum* parasite is required [20]. This study reports 5 non-synonymous and 2 synonymous random mutations among adequately responsive patients to ACT. There was a single non-synonymous mutation from a subset of the same group of patients reported earlier by Abdel Hamid et al. [23]. All these mutations were not linked with treatment failure. Other random mutations not linked to treatment failure from different parts of Africa were reported [44]. A recent report of molecular markers of resistance from different parts of Sudan not including the Blue Nile State, has indicated the absence of *Pfk13* mutation [16]. However, reports from Uganda and Equatorial Guinea have indicated the presence of resistant *Pfk13* gene mutations in these countries (*Pfk13* A675V and C580Y respectively) [45, 46]. Another single mutation in the *Pfk13* gene which might be associated with reduced clearance of parasites was reported from Ethiopia [47]. These reports are alarming for the malaria control programmes in Africa.

Mutant alleles of artemisinin resistance genetic background are shown in very low percentages in this study. Mutations of these genes are not directly linked to artemisinin resistance. However, they collectively lead to *Pfk13* gene mutation that can lead to failure of treatment [21].

## Conclusion

This study has shown that there is moderate resistance to chloroquine, very high resistance to SP and novel mutations in *Pfk13* gene not linked to artemisinin resistance. Absence of *exonuclease* mutations supports absence of PPQ resistance. This study supports the malaria treatment protocol currently used in Sudan.

## Supplementary information

**Additional file 1.** European Nucleotide Archive (ENA) accesion numbers of *P. falciparum* DNA samples successfully sequenced.

**Additional file 2.** Sequencing data of* Pfcrt*,* Pfmdr1*,* Pfdhfr*,* Pfdhps*,* Pfk13*,* exonuclease* molecular markers of resistance.

## Data Availability

The datasets used in this study are available as Additional file 2.
